# A comparative study of multiple physical assessment indicators to identify psychological symptoms: a cross-sectional study based on Chinese adolescents

**DOI:** 10.3389/fnut.2025.1511639

**Published:** 2025-03-04

**Authors:** Cunjian Bi, Xiaokang Ran, Feng Zhang, Yun Liu, Jun Li, Yintao Niu, Guangyan Yang

**Affiliations:** ^1^School of Physical Education, Chizhou University, Chizhou, China; ^2^College of Physical Education and Health, East China Normal University, Shanghai, China

**Keywords:** physical assessment, cross-sectional study, body roundness index, psychological symptoms, adolescents

## Abstract

**Background:**

Body mass index (BMI), waist circumference (WC), and waist-to-height ratio (WHtR) have long been important physical assessment indicators. In recent years a body shape index (ABSI) and body roundness index (BRI) have gained importance as new physical assessment indicators. However, it is unclear how these physical assessment indicators assess psychological symptoms in adolescents. The main purpose of this study was to determine the ability of BMI, WC, WHtR, ABSI, and BRI indicators to recognize psychological symptoms in Chinese adolescents and to examine whether ABSI and BRI are superior to the traditional BMI, WC, and WHtR.

**Methods:**

In this study, 42,472 (boys, 21,026, 49.5%) adolescents aged 12–18 years from six geographic regions in China were assessed for multiple physical assessment indicators (BMI, WC, WHtR, ABSI, BRI) and psychological symptoms from 2015 to 2016. One-way analysis of variance, Spearman’s rank test, multifactor logistic regression analysis, and ROC analysis were used to analyze the validity of multiple physical assessment indicators to identify psychological symptoms.

**Results:**

The prevalence of psychological symptoms among Chinese adolescents aged 12–18 years was 26.17%. The BMI, WC, WHtR, ABSI, and BRI of the adolescents were (20.19 ± 3.43) kg/m2, (69.68 ± 10.05) cm, (0.42 ± 0.06), (0.06 ± 0.01), and (2.05 ± 0.92), respectively. The results of Spearman’s rank test showed a positive correlation (*p* < 0.001) between BMI, WC, WHtR, and BRI were positively associated with psychological symptoms (*p* < 0.001). The highest Spearman correlation coefficient was found between WC and psychological symptoms (*r* = 0.134, *p* < 0.001), and BMI was the lowest (*r* = 0.108, *p* < 0.001). Overall, the prevalence of psychological symptoms for all five physical assessment indicators (BMI, WC, WHtR, ABSI, and BRI) showed an increasing trend from Q1 to Q4 (*p* < 0.001). Overall, WC (AUC: 0.61, 95%CI: 0.61–0.62), WHtR (AUC: 0.61, 95%CI: 0.60–0.61), and BRI (AUC: 0.61, 95%CI: 0.60–0.61) were highly and identically predictive of psychological symptoms; ABSI was the least predictive of psychological symptoms (AUC: 0.51, 95%CI: 0.50 ~ 0.51).

**Conclusion:**

This study found that neither the ABSI nor the BRI was superior to BMI, WC, or WHtR in predicting psychological symptoms in Chinese adolescents. The ABSI was the least predictive of psychological symptoms in Chinese adolescents, whereas the BRI showed potential as a surrogate for WC and WHtR indicators for assessing psychological symptoms. This study provides additional help and suggestions for better identification of psychological symptoms in Chinese adolescents.

## 1 Introduction

With changing lifestyles, lower levels of physical activity, increasing time spent in static behaviors and on screen, coupled with increased employment pressures, mental illness has become a serious public health problem and faces great challenges (1). According to statistics, about 450 million people worldwide suffer from various degrees of mental or behavioral disorders (2). Of these, more than 350 million people suffer from depression, and about 21 million from schizophrenia (3). In China alone, more than 100 million people suffer from mental illness, and in recent years there has been a trend toward a younger age group (4). The World Health Organization also notes that adolescence is a critical period for developing and maintaining social and emotional habits. About 10 to 20% of adolescents worldwide have varying degrees of psychological symptoms that are not adequately diagnosed and treated (5). Studies have also shown that half of adolescent psychiatric problems are first apparent by the age of 14, and 75% of these problems are manifested by age 24 (6). Some studies also show that the total prevalence of psychological symptoms among Chinese children and adolescents aged 6 ~ 16 years old is 17.5% (6). This shows that the occurrence of psychological symptoms among Chinese adolescents is particularly prominent and deserves attention and concern. Psychological symptoms in adolescents are mainly characterized by emotional instability, irritability, anxiety and even the onset of depressive symptoms, but also by abnormal behavior, as well as difficulties in adapting to the social environment and integrating into the classroom.

Studies have confirmed that there is a strong correlation between body mass index(BMI), waist circumference(WC), and waist-to-height ratio(WHtR) indicators, which reflect body shape or obesity, and various diseases and psychological symptoms (7, 8). The study showed that of all indicators, the best predictor of CM risk was WHtR, whereas a body shape index(ABSI) had the weakest associations with body fat, MetS, and CM risk (9). With regard to mental health, a study in a Spanish population confirmed the existence of a U-shaped relationship between BMI and psychological symptoms, suggesting that both low and high BMI have a negative impact on psychological symptoms (10). A study of college students also showed a u-curve relationship between body mass index and mood state, suggesting a strong link between the two (11). WC and WHtR are also closely associated with psychological symptoms. A study of Chinese adolescents confirmed that BMI and WHtR were positively associated with psychological stress, i.e., an increase in BMI and WHtR showed a co-development and change with an increase in psychological stress (12). The association between body composition and mental health may be due to factors such as discrimination against obese people brought about by physical obesity and disruption of hormone production in the body due to obesity (13). However, the results are not entirely consistent. A study of depressive symptoms in Chinese older adults found that no consistent trend in the prevalence of depressive symptoms and OR was observed across increasing WHR and WC (14). One study also showed that an increase in BMI was not independently associated with depression, which may provide some protection against anxiety, and no consistent trend in the prevalence of depressive symptoms and OR was observed with an increase in WC, suggesting that there is not a strong correlation between them (14).

As research continues, several indicators of body condition have emerged in recent years, such as the ABSI and the body roundness index(BRI). The ABSI is a new type of body condition indicator proposed by scholar Krakauer (15). ABSI was found to be positively associated with abdominal fat accumulation, and ABSI appeared to be superior to BMI and WC in predicting premature mortality (16, 17). However, the results are not consistent. Some studies have confirmed that ABSI is relatively weak in predicting cardiovascular disease compared with BMI (18). Another indicator, BRI, was developed by the team of scholar Thomas (19). BRI was found to be better at identifying cardiovascular disease (CVD) occurrence, but identification was not superior compared to BMI and WC (20). So far, the studies of ABSI and BRI have mainly focused on the association with various physiological indicators, while fewer studies have addressed the association between ABSI and BRI and psychological symptoms. However, it is unclear whether there are differences between these two new physical assessment indicators (ABSI, BRI) and BMI, WC, and WHtR in identifying psychological symptoms in Chinese adolescents. To this end, we assessed 42,472 adolescents aged 12–18 years from six regions of China on multiple physical indicators and psychological symptoms, aiming to analyze the ability of multiple physical indicators (BMI, WC, WHtR, ABSI, and BRI) to identify psychological symptoms in adolescents. Meanwhile, it was further tested whether ABSI and BRI were superior to traditional BMI, WC and WHtR for early identification and intervention of psychological symptoms in adolescents. The hypothesis of this study is that ABSI and BRI are superior to BMI, WC, and WHtR in recognizing psychological symptoms in adolescents.

## 2 Methods

### 2.1 Participants

**Figure 1:**
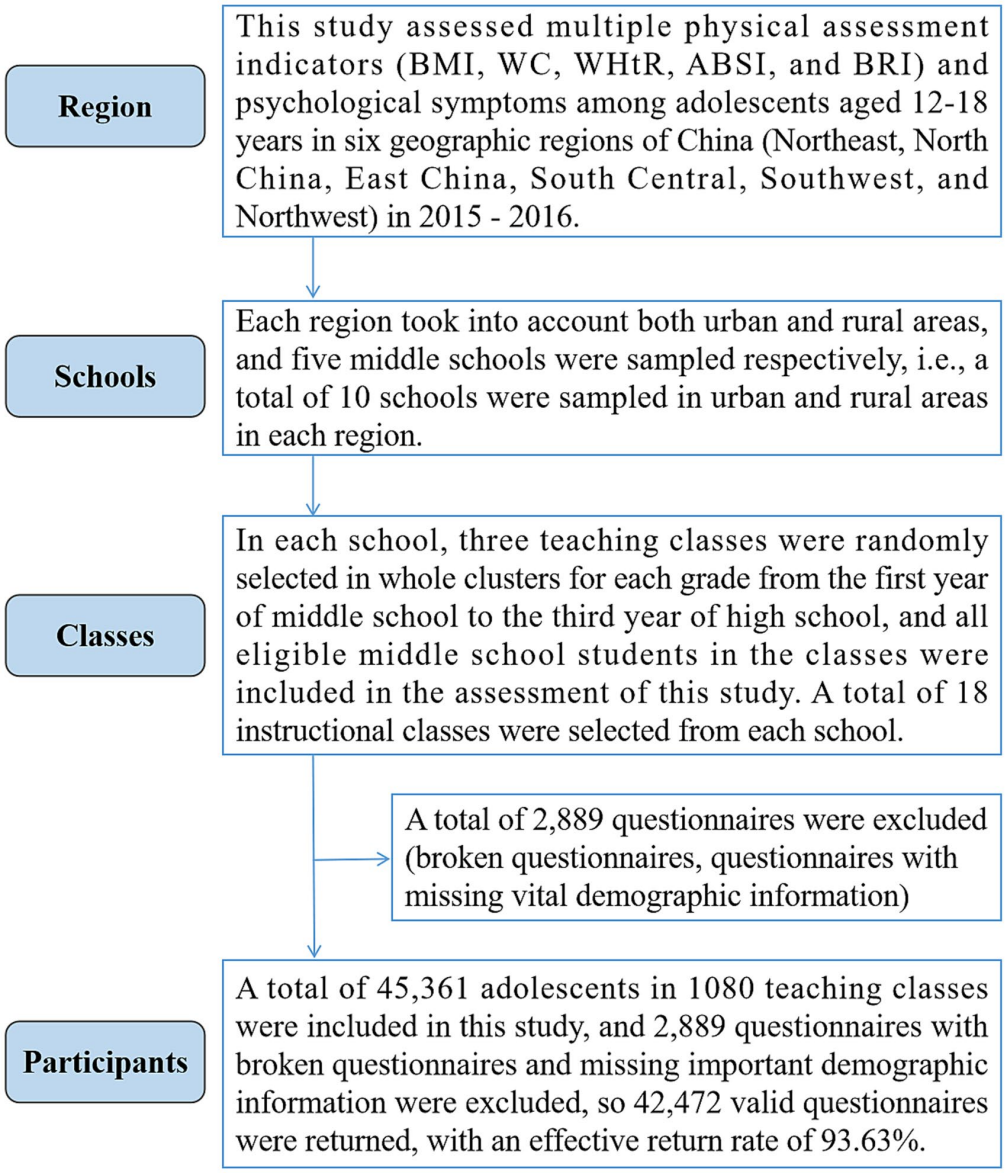
Flowchart of sampling Chinese adolescent participants.

In this study, 42,472 (boys, 21,026, 49.5%) adolescents aged 12–18 years old in six geographic regions of China (Northeast, North China, East China, Central-South, Southwest, and Northwest) were assessed for multiple physical assessment indicators and psychological symptoms from 2015 to 2016. The participant extraction process was divided into the following parts: First, according to the geographic regions of China, participant extraction was conducted in the six major regions of the country in order to guarantee a balanced distribution of the survey. Second, each region took into account both urban and rural areas and five middle schools were sampled respectively, i.e., a total of 10 schools were sampled in urban and rural areas in each region. Third, in each school, three teaching classes were randomly selected in whole clusters for each grade from the first year of middle school to the third year of high school, and all eligible middle school students in the classes were included in the assessment of this study. A total of 18 instructional classes were selected from each school. Participants were included on the condition that they were enrolled in middle school and that parents and participants gave voluntary informed consent to participate in the assessment. Finally, 45,361 adolescents in 1080 teaching classes were included in this study, and 2,889 questionnaires containing incomplete information due to fragmentation, missing important demographic information such as gender and age, and questionnaires with a response rate of less than 80% were excluded. so 42,472 valid questionnaires were returned, with an effective return rate of 93.63%. The specific sampling process of participants is shown in Figure 1.

This study was conducted in accordance with the Declaration of Helsinki. Informed consent was obtained from all subjects involved in the study and approved by the Human Ethics Committee of Chizhou University (202301015).

### 2.2 Multiple physical assessment indicators

Specific physical indicators included five indicators: BMI, WC, WHtR, ABSI, and BRI. BMI was assessed based on the participant’s height and weight results. It was weight (kg)/height (m)^2^. WC used a waist circumference tape for the measurements, which required the measurement of 1 cm above the participant’s navel, with the tape wrapped around the waist to record the size of the waist circumference in centimeters. The test results were accurate to 0.1 cm. Measurements of height, weight, and WC were assessed according to the test methods and instrumentation required by the China National Survey on Students’ Constitution and Health (CNSSCH). WHtR was waist circumference/height. ABSI was calculated using BMI and WC. The formula for ABSI is WC/(BMI2/3 × height1/2) (15). The BRI is calculated as 364.2–365.5*(1-[WC(m)/2π]^2^/[0.5*height(m)]^2^)½ (19).

### 2.3 Psychological symptoms

Psychological symptoms were assessed using participant self-assessment. The Multidimensional Sub-health Questionnaire of Adolescents (MSQA) was used to assess psychological symptoms in adolescents (21). The questionnaire consisted of 39 items, each of which was selected to correspond to a duration based on the actual situation of the participant in the past 6 months. Duration ranged from “none or less than 1 week” to “more than 3 months” on a scale of 6–1. Participants scored 0 points if they chose option 6, 5, or 4, and 1 point if they chose option 3, 2, or 1. The questionnaire consisted of three dimensions: emotional problems, behavioral problems, and social adjustment problems, each dimension consists of 18, 8, and 13 entries, respectively, and a positive result was indicated when the score was ≥3, ≥1, and ≥ 4, respectively. A score of ≥8 was judged as the presence of psychological symptoms. Total questionnaire score scores range from 0 to 39. The questionnaire is suitable for the assessment of the Chinese adolescent population and has been used in several studies (22–24). The questionnaire has good reliability and validity, with a Cronbach’ s *α* coefficient of 0.854 (25).

### 2.4 Covariates

The oblique variables in this study include place of residence, father’s education, mother’s education, monthly household income, and sugar-sweetened beverages (SSBs) indicators. Place of residence includes large and medium-sized cities, small cities, and the countryside. Cities are categorized according to their population size. Large and medium-sized cities are classified according to the population size of the city. Large and medium-sized cities have a population of more than 1 million; large cities have a population of 500,000-1,000,000; medium-sized cities have a population of 200,000-500,000; and small cities have a population of less than 200,000. In this study, large and medium-sized cities are defined as cities with a population of more than 200,000, and small cities are defined as cities with a population of less than 200,000. Countryside is defined as the participants’ household registration in rural areas. Father’s education and mother’s education are classified into father’s education and mother’s education was categorized as primary school, middle school, high school, and college or above; monthly household income was categorized as <2000, 2001–5,000, 5,001–8,000, and > 8,000; SSB consumption was categorized into ≥5 times/week, 3–4 times/week, and ≤ 2 times/week. Participants were categorized according to their SSB consumption in the past 1 month. Each consumption was calculated as 330 mL (26).

### 2.5 Quality control

Participants were asked to wear as light clothing as possible and remove their shoes before being assessed for height, weight, and WC. Participants were asked to empty their bowels and urine before the assessment. The assessors calibrated the assessment instruments daily to guarantee the accuracy of the assessments. Psychological symptoms were assessed using a paper-based questionnaire. Staff explained the purpose and requirements of the assessment to participants, and the questionnaires were distributed and returned on the spot. The questionnaires were distributed and collected on the spot. Participants filled out the questionnaires independently according to their actual situation. Participants can ask the staff if they have any questions during the assessment process.

### 2.6 Statistical analysis

The results of participants’ general characteristics were presented using percentages (%) and means (standard deviations). Correlations between psychological symptoms and BMI, WC, WHtR, ABSI, and BRI were analyzed using the Spearman rank test. The chi-square test was used to compare differences in the detection rates of psychological symptoms in adolescents categorized by quartiles of different BMI, WC, WHtR, ABSI, and BRI aspects. Comparisons of BMI, WC, WHtR, ABSI, and BRI in adolescents with or without psychological symptoms were presented and compared using continuous variables. Associations between BMI, WC, WHtR, ABSI, and BRI and psychological symptoms in adolescents were analyzed using multifactor logistic regression analysis. The presence of psychological symptoms was used as the dependent variable, and BMI, WC, WHtR, ABSI, and BRI were used as independent variables adjusted for age, Place of residence, Father’s education, Mother’s education, Monthly household income, and SSBs. BMI, WC, WHtR, ABSI, and BRI were divided into Q1, Q2, Q3, and Q4 groups according to the quartiles of age and gender, and multifactorial logistic regression analyses were performed with the Q1 group as the reference, and ORs and 95% CIs were reported, respectively. The ability or validity of five physical assessment indicators (BMI, WC, WHtR, ABSI, and BRI) to identify psychological symptoms was assessed using ROC analysis based on the area under the curve (AUC) and 95% confidence interval (CI). Data processing and analysis were performed using SPSS 25.0 software. Statistical significance was considered to exist at *p* < 0.05.

## 3 Results

In this study, 42,472 (boys 21,026, 49.5%) participants were assessed for several physical measures and psychological symptoms. The mean age of the participants was (15.12 ± 1.88) years. The prevalence of psychological symptoms among participants was 26.17% (11,113/42472). Participants’ BMI, WC, WHtR, ABSI, and BRI were (20.19 ± 3.43) kg/m2, (69.68 ± 10.05) cm, (0.42 ± 0.06), (0.06 ± 0.01), (2.05 ± 0.92) respectively.

Table 1 shows that the differences in the prevalence of psychological symptoms were statistically significant when participants were compared across gender, Place of residence, Father’s education, Mother’s education, Monthly household income, and SSBs (*p* < 0.01). The difference between adolescents with and without psychological symptoms was also statistically significant (*p* < 0.001) when comparing the prevalence of psychological symptoms in terms of Height, Weight, BMI, WC, WHtR, ABSI, and BRI.

**Table 1:** Comparison of participants’ general demographic characteristics psychological symptoms.

Categorization	Psychological symptoms or not		Total	*p*-value
	No	Yes		
N	31,359 (73.83)	11,113 (26.17)	42,472	<0.001
Age	15.13 ± 1.9	15.10 ± 1.83	15.12 ± 1.88	0.160
Sex				<0.001
Boys	15,049 (48.0)	5,977 (53.8)	21,026 (49.5)	
Girls	16,310 (52.0)	5,136 (46.2)	21,446 (50.5)	
Place of residence				0.002
Large and medium-sized cities	12,529 (40.0)	4,252 (38.3)	16,781 (39.5)	
Small city	11,215 (35.8)	4,004 (36.0)	15,219 (35.8)	
Countryside	7,615 (24.3)	2,857 (25.7)	10,472 (24.7)	
Father’s education				<0.001
Primary school	3,505 (11.2)	1,420 (12.8)	4,925 (11.6)	
Middle school	11,084 (35.3)	3,977 (35.8)	15,061 (35.5)	
High school	10,855 (34.6)	3,648 (32.8)	14,503 (34.1)	
College or above	5,915 (18.9)	2068 (18.6)	7,983 (18.8)	
Mother’s education				0.001
Primary school	5,438 (17.3)	2076 (18.7)	7,514 (17.7)	
Middle school	10,713 (34.2)	3,861 (34.7)	14,574 (34.3)	
High school	10,226 (32.6)	3,485 (31.4)	13,711 (32.3)	
College or above	4,982 (15.9)	1,691 (15.2)	6,673 (15.7)	
Monthly household income				<0.001
< 2000	3,536 (11.3)	1,560 (14.0)	5,096 (12.0)	
2001–5,000	11,065 (35.3)	3,923 (35.3)	14,988 (35.3)	
5,001–8,000	9,718 (31.0)	3,150 (28.3)	12,868 (30.3)	
>8,000	7,040 (22.4)	2,480 (22.3)	9,520 (22.4)	
SSBs				<0.001
≥ 5 times/week	4,303 (13.7)	2038 (18.3)	6,341 (14.9)	
3–4 times/week	16,573 (52.8)	5,492 (49.4)	22,065 (52.0)	
≤ 2 times/week	10,483 (33.4)	3,583 (32.2)	14,066 (33.1)	
Height	165.28 ± 8.82	166.14 ± 9.15	165.50 ± 8.91	<0.001
Weight	54.67 ± 11.36	58.3 ± 13.51	55.62 ± 12.07	<0.001
BMI	19.9 ± 3.19	20.99 ± 3.91	20.19 ± 3.43	<0.001
Waist circumference	68.45 ± 9.04	73.14 ± 11.78	69.68 ± 10.05	<0.001
WHtR	0.41 ± 0.05	0.44 ± 0.07	0.42 ± 0.06	<0.001
ABSI	0.06 ± 0.01	0.06 ± 0.01	0.06 ± 0.01	<0.001
BRI	1.94 ± 0.78	2.39 ± 1.17	2.05 ± 0.92	<0.001
Emotional symptoms	0.62 ± 1.16	7.48 ± 3.61	2.42 ± 3.67	<0.001
Behavioral symptoms	0.13 ± 0.44	2.73 ± 2.05	0.81 ± 1.60	<0.001
Social adaptation difficulties	0.45 ± 0.94	5.19 ± 2.96	1.69 ± 2.70	<0.001
Psychological symptoms	1.21 ± 1.93	15.4 ± 6.82	4.92 ± 7.34	<0.001

N, Numbers. SSBs, Sugar-sweetened beverage. BMI, body mass index; WHtR, waist-to-height ratio; ABSI, a body shape index; BRI, body roundness index.

Table 2 shows the results of Spearman’s rank test for MULTIPLE physical assessment indicators (BMI, WC, WHtR, ABSI, BRI) and psychological symptoms. The overall results showed that there was a positive correlation association between BMI, WC, WHtR, BRI, and psychological symptoms in Chinese adolescents (*p* < 0.001). The highest Spearman’s correlation coefficient was between WC and psychological symptoms (*r* = 0.134, *p* < 0.001), and the lowest was between BMI and psychological symptoms (*r* = 0.108, *p* < 0.001).

**Table 2:** Spearman’s rank test for multiple physical assessment indicators (BMI, WC, WHtR, ABSI, BRI) and psychological symptoms in Chinese youths.

Sex	BMI	WC	WHtR	ABSI	BRI
Boys (*n* = 21,026)					
Psychological symptoms	0.109	0.129	0.126	−0.003	0.126
*P*-value	<0.001	<0.001	<0.001	0.625	<0.001
Girls (*n* = 21,446)					
Psychological symptoms	0.101	0.123	0.126	0.011	0.126
*P*-value	<0.001	<0.001	<0.001	0.099	<0.001
Total (*n* = 42,472)					
Psychological symptoms	0.108	0.134	0.129	0.002	0.129
*P*-value	<0.001	<0.001	<0.001	0.616	<0.001

BMI, body mass index; WC, waist circumference; WHtR, waist-to-height ratio; ABSI, a body shape index; BRI, body roundness index.

Table 3 shows the prevalence of psychological symptoms in the BMI, WC, WHtR, ABSI, and BRI quartiles of Chinese adolescents. Overall, the prevalence of psychological symptoms in all five physical assessment indicators (BMI, WC, WHtR, ABSI, and BRI) showed an increasing trend from Q1 to Q4 (*p* < 0.001). The prevalence of psychological symptoms increased from 20.3 to 36.3% in Chinese adolescents with BMI from Q1 to Q4, and from 18.9 to 38.1% in WC from Q1 to Q4. WHtR in Q1 to Q4, the detection rate of psychological symptoms increased from 18.8 to 35.6%. ABSI in Q1 to Q4, the detection rate of psychological symptoms increased from 26.0 to 29.1%. For BRI from Q1 to Q4, the detection rate of psychological symptoms increased from 18.9 to 35.7%. The same trend was observed for both male and female students from Q1 to Q4 (*p* < 0.001).

**Table 3:** Prevalence of psychological symptoms among Chinese adolescents in quartiles of BMI, WC, WHtR, ABSI, and BRI (%).

Quartile	BMI	WC	WHtR	ABSI	BRI
Boys					
Q1 [*n*, (%)]	1,084 (21.9)	767 (19.9)	928 (19.5)	1,591 (28.7)	919 (19.6)
Q2 [*n*, (%)]	1,061 (22.6)	820 (21.2)	1,088 (22.7)	1,570 (28.4)	1,125 (22.8)
Q3 [*n*, (%)]	1,457 (28.4)	1826 (28.9)	1735 (32.6)	1,243 (25.7)	1713 (32.6)
Q4 [*n*, (%)]	2,375 (38.1)	2,564 (36.6)	2,226 (36.2)	1,573 (30.6)	2,220 (36.2)
*P*-value	<0.001	<0.001	<0.001	<0.001	<0.001
Girls					
Q1 [*n*, (%)]	1,053 (18.9)	1,347 (18.4)	1,039 (18.2)	1,097 (22.8)	1,024 (18.2)
Q2 [*n*, (%)]	1,188 (20)	1,265 (21.2)	1,027 (18.3)	1,404 (24.1)	1,060 (18.3)
Q3 [*n*, (%)]	1,397 (25.4)	1,353 (25.4)	1,457 (26.5)	1,214 (21.4)	1,445 (26.5)
Q4 [*n*, (%)]	1,498 (33.8)	1,171 (41.7)	1,613 (34.8)	1,421 (27.6)	1,607 (35.1)
*P*-value	<0.001	<0.001	<0.001	<0.001	<0.001
Total					
Q1 [*n*, (%)]	2,137 (20.3)	2,114 (18.9)	1967 (18.8)	2,688 (26.0)	1943 (18.9)
Q2 [*n*, (%)]	2,249 (21.1)	2085 (21.2)	2,115 (20.3)	2,974 (26.2)	2,185 (20.4)
Q3 [*n*, (%)]	2,854 (26.8)	3,179 (27.3)	3,192 (29.5)	2,457 (23.4)	3,158 (29.5)
Q4 [*n*, (%)]	3,873 (36.3)	3,735 (38.1)	3,839 (35.6)	2,994 (29.1)	3,827 (35.7)
*P*-value	<0.001	<0.001	<0.001	<0.001	<0.001

BMI, body mass index; WC, waist circumference; WHtR, waist-to-height ratio; ABSI, a body shape index; BRI, body roundness index.

Table 4 shows the logistic regression analysis of BMI, WC, WHtR, ABSI, and BRI with psychological symptoms in Chinese adolescents. Psychological symptoms were used as the dependent variable (Yes = 1, No = 0), and BMI, WC, WHtR, ABSI, and BRI were used as the independent variables, respectively, with age, Place of residence, Father’s education, Mother’s education, Monthly household income, the SSBs were adjusted for Logistic regression analysis. Overall results showed that adolescents in the BMI for the Q4 group (OR = 3.32, 95% CI: 2.94 ~ 3.74) had a higher risk of developing psychological symptoms (*p* < 0.001) compared to the Q1 group as the reference group. WC was a higher risk of psychological symptoms in adolescents in the Q4 group (OR = 1.62, 95% CI: 1.42 ~ 1.85) (*p* < 0.001). Adolescents in the ABSI as Q4 group (OR = 2.40, 95%CI: 2.15 ~ 2.67) also had a higher risk of developing psychological symptoms (*p* < 0.001).

**Table 4:** Logistic regression analysis of BMI, WC, WHtR, ABSI, and BRI with psychological symptoms in Chinese adolescents [OR, (95%CI)].

Quartile	BMI	WC	WHtR	ABSI	BRI
Boys					
Q1 (Reference)	1.00	1.00	1.00	1.00	1.00
Q2 [OR(95%CI)]	1.27 (1.13 ~ 1.41)^c^	1.01 (0.89 ~ 1.15)	1.60 (0.78 ~ 3.27)	1.25 (1.14 ~ 1.37)^c^	0.55 (0.27 ~ 1.12)
Q3 [OR(95%CI)]	1.97 (1.74 ~ 2.25)^c^	1.23 (1.06 ~ 1.43)^b^	2.52 (1.08 ~ 5.88)	1.42 (1.27 ~ 1.59)^c^	0.41 (0.18 ~ 0.97)^a^
Q4 [OR(95%CI)]	3.64 (3.09 ~ 4.29)^c^	1.36 (1.13 ~ 1.63)^c^	1.82 (0.51 ~ 6.54)	2.44 (2.11 ~ 2.82)^c^	0.43 (0.12 ~ 1.56)
Girls					
Q1 (Reference)	1.00	1.00	1.00	1.00	1.00
Q2 [OR(95%CI)]	1.33 (1.19 ~ 1.48)^c^	1.01 (0.9 ~ 1.14)	1.01 (0.57 ~ 1.76)	1.35 (1.22 ~ 1.50)^c^	0.80 (0.46 ~ 1.40)
Q3 [OR(95%CI)]	2.00 (1.76 ~ 2.28)^c^	1.00 (0.85 ~ 1.16)	0.99 (0.46 ~ 2.13)	1.42 (1.25 ~ 1.61)^c^	1.04 (0.48 ~ 2.23)
Q4 [OR(95%CI)]	3.02 (2.53 ~ 3.60)^c^	1.88 (1.54 ~ 2.30)^c^	0.31 (0.10 ~ 0.98)^a^	2.33 (1.99 ~ 2.73)^c^	2.57 (0.81 ~ 8.11)
**Total**					
Q1 (Reference)	1.00	1.00	1.00	1.00	1.00
Q2 [OR(95%CI)]	1.30 (1.20 ~ 1.40)^c^	1.02 (0.94 ~ 1.11)	1.22 (0.78 ~ 1.89)	1.29 (1.20 ~ 1.38)^c^	0.69 (0.44 ~ 1.07)
Q3 [OR(95%CI)]	1.98 (1.81 ~ 2.17)^c^	1.17 (1.06 ~ 1.30)^b^	1.51 (0.87 ~ 2.64)	1.41 (1.30 ~ 1.54)^c^	0.64 (0.37 ~ 1.13)
Q4 [OR(95%CI)]	3.32 (2.94 ~ 3.74)^c^	1.62 (1.42 ~ 1.85)^c^	0.61 (0.27 ~ 1.41)	2.40 (2.15 ~ 2.67)^c^	1.21 (0.52 ~ 2.80)

BMI, body mass index; WC, waist circumference; WHtR, waist-to-height ratio; ABSI, a body shape index; BRI, body roundness index.

a *P < 0.05.*

b *P < 0.01.*

c *P < 0.001.*

Table 5 shows the AUC and 95% CI results of the human assessment indicators (BMI, WC, WHtR, ABSI, BRI) in predicting psychological symptoms. Overall, WC (AUC: 0.61, 95%CI: 0.61–0.62), WHtR (AUC: 0.61, 95%CI: 0.60–0.61), and BRI (AUC: 0.61, 95%CI: 0.60 ~ 0.61) were highly and equally predictive of psychological symptoms. ABSI had the lowest predictive effect on psychological symptoms (AUC: 0.51, 95%CI: 0.50 ~ 0.51).

**Table 5:** Area under the curve of BMI, WC, WHtR, ABSI, BRI, and psychological symptoms in Chinese adolescents.

Index	Boys (*n* = 21,026)	Girls (*n* = 21,446)	Total (*n* = 42,472)
BMI	0.59 (0.58 ~ 0.60)	0.58 (0.57 ~ 0.59)	0.59 (0.58 ~ 0.59)
WC	0.61 (0.60 ~ 0.62)	0.60 (0.59 ~ 0.61)	0.61 (0.61 ~ 0.62)
WHtR	0.61 (0.60 ~ 0.61)	0.60 (0.59 ~ 0.61)	0.61 (0.60 ~ 0.61)
ABSI	0.50 (0.49 ~ 0.51)	0.52 (0.51 ~ 0.52)	0.51 (0.50 ~ 0.51)
BRI	0.61 (0.60 ~ 0.61)	0.60 (0.59 ~ 0.61)	0.61 (0.60 ~ 0.61)

BMI, body mass index; WC, waist circumference; WHtR, waist-to-height ratio; ABSI, a body shape index; BRI, body roundness index.

**Figure 2:**
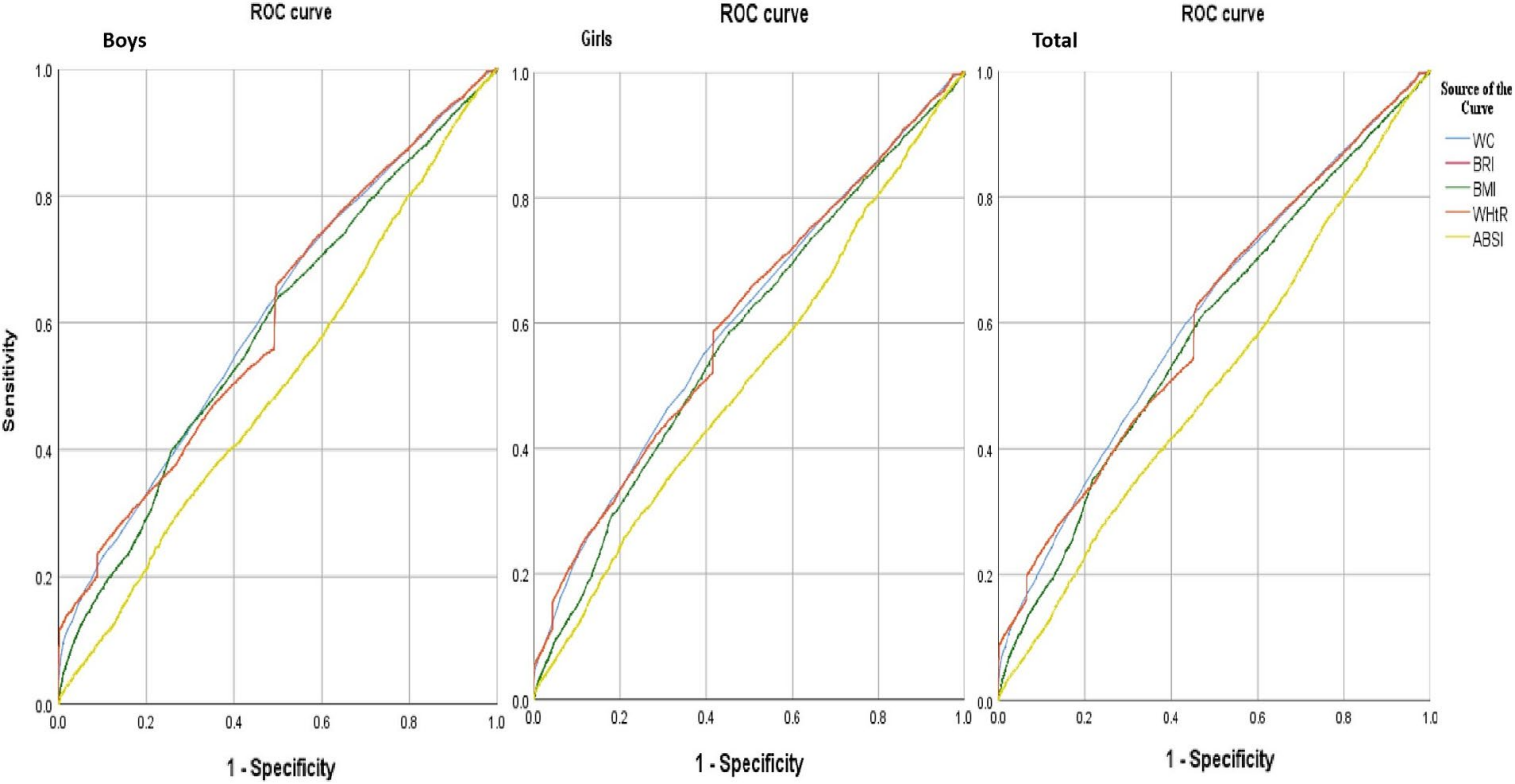
ROC curves of the ability of Chinese adolescents’ BMI, WC, WHtR, ABSI, and BRI to discriminate between the presence and absence of psychological symptoms. BMI, body mass index, WC, waist circumference, WHtR, waist-to-height ratio, ABSI, a body shape index, BRI, body roundness index.

Figure 2 shows the trend of AUC of physical assessment indicators (BMI, WC, WHtR, ABSI, BRI) in predicting psychological symptoms in Chinese adolescents. Overall, it can be seen that the area under the curve of ABSI was lowest for boys and girls.

## 4 Discussion

In this cross-sectional study, we evaluated the validity of MULTIPLE physical assessment indicators (BMI, WC, WHtR, ABSI, BRI) to identify psychological symptoms in order to better screen for psychological symptoms in Chinese adolescents. The results of this study showed that after adjusting for age, Place of residence, Father’s education, Mother’s education, Monthly household income, and SSBs, the WC, WHtR, and BRI were highly and identically psychologically symptomatic for Chinese adolescents predictive effects, with an AUC of 0.61. However, ABSI had the lowest predictive effect on psychological symptoms in Chinese adolescents with an AUC of 0.51, followed by BMI with an AUC of 0.59. This shows that ABSI and BMI are not suitable for identifying psychological symptoms in Chinese adolescents.BRI, together with WC and WHtR, can better identify psychological symptoms in Chinese adolescents.

Thomas et al. first researched and developed the BRI index to assess the amount of body fat in people as well as visceral fat content (19). There are relatively few studies in the current research addressing the association of BRI with physical health indicators compared to BMI. A study of BRI recognition of CVD and diabetes mellitus (DM) confirmed that BRI was able to better recognize CVD, but was weaker in recognizing DM compared to WC and WHtR (20). Another national cohort study of U.S. adults found that the BRI is trending upward from year to year and, more importantly, found a U-shaped relationship between the BRI and all-cause mortality, with adults with a BRI of less than 3.4 having a 25% increased risk, suggesting that the BRI is a better identifier of all-cause mortality in U.S. adults. This study will provide strong evidence in favor of the use of the BRI as a non-invasive screening indicator for mortality risk assessment (27). With the deepening of research, the BRI index has gradually been paid attention to by Chinese scholars. A study of ABSI and BRI in identifying diabetes in a rural population in northeastern China found that neither ABSI nor BRI was superior to BMI, WC, or WHtR, with WHtR being the strongest predictor of diabetes and ABSI the weakest predictor, and that BRI showed potential as an alternative indicator of obesity to assess diabetes (28). However, previous studies have mainly focused on the prediction and identification of BRI on various physical diseases, while fewer studies have been conducted on the prediction and identification of BRI and psychological aspects, and no studies have been found on the identification of BRI on psychological symptoms in Chinese adolescents (29, 30). A cross-sectional study using data from the U.S. National Health and Nutrition Examination Survey (NHANES) 2011–2018 confirmed that after adjusting for age, gender, race/ethnicity, educational attainment, marital status, poverty-to-income ratio, alcohol status, smoking status, hypertension, diabetes, cardiovascular disease, energy intake, physical activity, total cholesterol, and triglycerides, BRI levels showed a significant positive correlation with increased prevalence of depression in U.S. adults, and the study also confirmed that the BRI can be used as a simple anthropometric index to predict depression in U.S. adults, a study that provides strong support for the BRI to better identify mental health (31). Another study also confirmed a positive correlation between BRI and depression in US adults, mediated by the Atherogenic Index of Plasma (AIP), a study that also provides strong evidence of the association between BRI and depression and could be incorporated into depression screening as a non-invasive indicator for identifying depression (32). Although some shortcomings of the BRI have been demonstrated in previous studies, the BRI was able to better assess the percentage of body fat of the participants compared to BMI and ABSI, and thus better predict psychological symptoms in Chinese adolescents.

To the best of our knowledge, the present study is the first to analyze and analyze BMI, WC, WHtR, ABSI, and BRI for identifying psychological symptoms in Chinese adolescents and confirmed that BRI, like previous commonly used indexes, WC and WHtR, are able to identify psychological symptoms of Chinese adolescents better. However, it is worth noting that the present study confirmed that ABSI and BMI are low in identifying psychological symptoms in Chinese adolescents. The reason may be because BMI only reflects the external body shape or proportionality of the body, while it does not reflect the internal body fat content of the body well enough to identify hidden obese individuals (33, 34). On the contrary, people with hidden obesity are also an important group for the occurrence of psychological problems, which need to be paid enough attention and concern (35). Relatively more studies have been conducted on ABSI and health. Studies have confirmed that ABSI is independent of height, weight, and BMI, and have also found that ABSI is a better predictor of risk of premature death compared to BMI and WC, suggesting that ABSI has a positive effect on predicting the risk of premature death (15). It has also been shown that the ABSI is a more easily calculated dynamic indicator and that the ABSI’s prediction of mortality risk has gender and age sensitivities across BMI types, and can be used in clinical decision-making and in the prediction of lifestyle and other health risks (36). However, the results of the ABSI in predicting health are not consistent. Some studies have shown that the ABSI has no significant effect on predicting cardiovascular disease, suggesting that this indicator is ineffective in predicting the occurrence of cardiovascular disease (20). Other studies have confirmed that the ABSI has not been found to be a better predictor of diabetes and hypertension compared to BMI and WC, suggesting that the ABSI is not the best predictor of diabetes or hypertension (37). In addition, a 15-year follow-up study of Chinese adults found that the ABSI, WC, and BMI were relatively consistent in their ability to predict diabetes, a finding that suggests that the ABSI was not found to be a better predictor of diabetes compared with BMI and WC (38). The results of this study also showed that the ABSI did not better predict the occurrence of psychological symptoms in Chinese adolescents, suggesting that the ABSI should be used with caution in predicting physical and mental health and that more research should be conducted on the relationship between the ABSI and various physical and mental health indicators in the future. In addition, the low predictive utility of the ABSI for psychological symptoms may be due to the fact that the ABSI was initially developed as a predictor of mortality risk in a follow-up study and is not optimal for predicting psychological symptoms. Furthermore, as with the results of related studies, we believe that the predictive results of the ABSI were not consistent across studies, mainly related to differences in the countries, ages, and ethnicities investigated in the different studies (37). It is worth noting that although there is some discrepancy in the ABSI’s ability to predict psychological and certain health problems, and the reasons for this discrepancy need to be further identified, the ABSI is defined as a separate indicator independent of BMI, and therefore can be analyzed as an important complementary indicator when identifying subjects at risk for certain diseases.

There are certain strengths and limitations of this study that need to be explained. In terms of the strengths of the study, First, this study is the first to analyze the research on the identification of psychological symptoms in Chinese adolescents by BMI, WC, WHtR, ABSI, and BRI indicators, and it was found that BRI, like the previous commonly used indicators, WC, and WHtR, are better able to identify psychological symptoms in Chinese adolescents, and that this finding can play a positive role in the prediction of the occurrence of psychological symptoms in Chinese adolescents and the intervention of such symptoms. Second, the sample size of participants in this study is large and distributed in six regions of China, and the distribution of participants is broad, which is representative and typical. However, this study also has certain limitations. First, initially, the ABSI was developed to predict the risk of death, while the present study was used for the prediction of psychological symptoms in adolescents, which may be an important reason why the ABSI, unlike the BRI, failed to better predict psychological symptoms in adolescents. The effectiveness of these indicators in predicting psychological symptoms in different groups should be further explored in the future. Second, the ABSI and BRI indicators were initially developed based on studies of population characteristics in Western countries or the United States, and are not applicable to the Chinese population. In the future, certain adjustments or modifications should be made based on different studies to better fit the characteristics of the Chinese population and to improve the prediction effect. Finally, the present study was a cross-sectional study that was only able to analyze the associations that existed between BMI, WC, WHtR, ABSI, BRI, and psychological symptoms, but not the causal associations that existed between them. Future cohort studies should be conducted to analyze the causal associations that exist between them.

## 5 Conclusion

In conclusion, this study found that WC, WHtR, and BRI were highly and equally predictive of psychological symptoms in Chinese adolescents, while ABSI was the least predictive of psychological symptoms in Chinese adolescents, followed by BMI. This shows that ABSI and BMI should be combined with more indicators in identifying psychological symptoms in Chinese adolescents. In addition, BRI, like WC and WHtR, can be used as an alternative indicator to identify psychological symptoms in Chinese adolescents. In future studies, traditional body size indicators should be used in conjunction with new ones, and more extensive research and exploration should be conducted on the strengths and weaknesses of body size indicators in recognizing psychological symptoms for different groups of Chinese adolescents.

## Data Availability

The raw data supporting the conclusions of this article will be made available by the authors, without undue reservation.
